# Modeling Seasonal Variations in Vertical GPS Coordinate Time Series Using Independent Component Analysis and Varying Coefficient Regression

**DOI:** 10.3390/s20195627

**Published:** 2020-10-01

**Authors:** Bin Liu, Xuemin Xing, Jianbo Tan, Qing Xia

**Affiliations:** 1 Engineering Laboratory of Spatial Information Technology of Highway Geological Disaster Early Warning in Hunan Province, Changsha University of Science & Technology, Changsha 410114, China; xuemin.xing@csust.edu.cn (X.X.); tanjianbo@imde.ac.cn (J.T.); xiaqingfriendxia@126.com (Q.X.); 2 School of Traffic and Transportation Engineering, Changsha University of Science and Technology, Changsha 410014, China

**Keywords:** vertical GPS coordinate time series, spatiotemporal modeling, independent component analysis, varying coefficient regression

## Abstract

Common seasonal variations in Global Positioning System (GPS) coordinate time series always exist, and the modeling and correction of the seasonal signals are helpful for many geodetic studies using GPS observations. A spatiotemporal model was proposed to model the common seasonal variations in vertical GPS coordinate time series, based on independent component analysis and varying coefficient regression method. In the model, independent component analysis (ICA) is used to separate the common seasonal signals in the vertical GPS coordinate time series. Considering that the periodic signals in GPS coordinate time series change with time, a varying coefficient regression method is used to fit the separated independent components. The spatiotemporal model was then used to fit the vertical GPS coordinate time series of 262 global International GPS Service for Geodynamics (IGS) GPS sites. The results show that compared with least squares regression, the varying coefficient method can achieve a more reliable fitting result for the seasonal variation of the separated independent components. The proposed method can accurately model the common seasonal variations in the vertical GPS coordinate time series, with an average root mean square (RMS) reduction of 41.6% after the model correction.

## 1. Introduction

Seasonal signals are always observed in Global Positioning System (GPS) coordinate time series [1], which are thought to be a combination of different factors. The main causes of the seasonal signals in vertical GPS coordinate time series are thought to be the uncorrected mass loading deformations, usually including atmospheric loading [2,3], hydrological loading [4,5,6] and non-tidal loading [7,8]. The effects of different mass loadings vary in different regions due to differences in environmental change. Another factor that causes the seasonal variations in GPS time series is the false periodic signals generated in GPS processing, e.g., the errors in satellite orbit, tropospheric delay, antenna phase center correction models and multipath effects [1,9,10,11]. In addition, the thermal expansion effect of the bedrock is also thought to be one of the reasons for the periodic changes of the vertical GPS coordinates time series [12].

The existence of the seasonal variations in GPS time series will affect the estimation of motion velocities and their uncertainties on sites [13,14], which is not conducive to the study of geophysical applications using GPS, e.g., assessment of glacial isostatic adjustment (GIA) models using vertical GPS observations [15]. Thus, it is very necessary to model or separate the seasonal signals in GPS coordinate time series for many geodetic studies, especially in the vertical direction [1,9,10,16].

An optional measure to estimate the seasonal variations is to quantify the potential periodic signals based on their physical sources. For example, the mass loading displacements can be calculated by convolving the grid surface mass data with Green’s functions [17], and currently, various environmental loading products are available for estimating the mass loading effects [9,11,18,19]. The estimation results would be different due to differences in the Green’s functions, the references frame of the Green’ functions, the reference mass model, and the land–ocean mask [9]; in many studies, the effect of correcting the seasonal variations in GPS observations using mass loading models is limited, due to the complexity of the Earth’s structure and the spatial resolution of grid mass data. 

The other idea to estimate the seasonal signals in GPS coordinate time series, is modeling the potential period changes using mathematical fitting methods. The most classic approach is using Least-squares with sine and cosine functions for a specific period [20], prior to—or simultaneous with—some noise assumption [21,22,23]. However, only constant amplitudes and phases can be estimated using this fitting method, which cannot fully describe the actual periodic changes in many cases. True seasonal variations in GPS coordinate time series are not constant from year to year [24], and several approaches have been proposed to model the time-varying seasonal signals, such as non-parametric annual signal [25], Kalman filter techniques [26], piecewise continuous linear polynomials [27], singular spectrum analysis (SSA) [24], ensemble empirical mode decomposition (EEMD) [28], and so on. All the methods above are aimed at the GPS time series at a single GPS site, and are not conducive to the overall analysis of regional GPS observations. Actually, the seasonal signals at regional GPS sites have similar features [29,30]. Gruszczynska et al. [30] used multichannel singular spectrum analysis (MSSA) to model the common seasonal signals in GPS observations in different regions around the world and without influencing the high frequency part of the spectra. Some other data-driven mathematical methods have been proposed to be used in modeling the geophysical signals from regional geodetic time series, e.g., principle component analysis (PCA) and independent component analysis (ICA), which have been widely used in smoothing and modeling GPS time series [15,23,31,32,33,34,35,36,37]. ICA has especially been widely used in the separation of potential sources of seasonal signals in vertical GPS coordinate time series, and the main independent components (ICs) were thought to be consistent with some of the geophysical signals [32,33,34]. 

In this paper, we conducted a spatiotemporal analysis of vertical GPS coordinates on a global scale with ICA, separating the common seasonal components of all the sites. Further, a varying coefficient (VC) regression method was used to model the separated common seasonal signals. Combining the spatial responses (SRs) achieved in ICA processing, we built a spatiotemporal model to analyze the seasonal signals in all the GPS sites in the case study.

## 2. Methods

### 2.1. Spatiotemporal Model Using ICA and VC Regression

**Figure 1:**
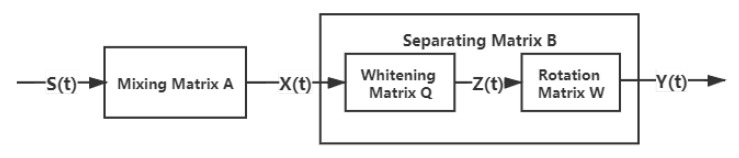
Process of independent component analysis.

ICA is a blind source separation method that can separate statistically independent signals from mixed signals using the higher order statistical characteristics of the source signals [38]. The process of ICA is illustrated in Figure 1.

Each observation $X\left(t\right)={\left[X_{1}\left(t\right),X_{2}\left(t\right),\cdots ,X_{n}\left(t\right)\right]}^{T}$ can be viewed as the mixture of the source signals $\;S\left(t\right)={\left[S_{1}\left(t\right),S_{2}\left(t\right),\cdots ,\;\;S_{n}\left(t\right)\right]}^{T}$ with different weights. The purpose of ICA is to obtain a separating matrix  B, then the signals  Y(t), the best estimates of S(t). Generally, the process of ICA can be divided into two steps. The first is to whiten the observed variables by Z=QX and $E\left(ZZ^{T}\right)=I$, where Q is called the whitening matrix. The second step is to obtain a rotation matrix W by following the ICA algorithm, then we obtain the $Y=W^{T}Z$. The row vectors in Y are ICs to be estimated and the column vectors in the inverse matrix of $W^{T}$ are the spatial resonses of ICs. To obtain each weight vector w in the rotation matrix W, FastICA algorithm based on negentropy [39,40] was used in ICA analysis. The estimation process of W is as follows:

(1) Centralize and whiten the observed data, obtain the whitened data Z;
(2) Choose an initial weight vector of unit norm (random) w;
(3) Update $w^{+}$ by $w^{+}=E\left[Zg\left(w^{T}z\right)\right]-E\left[g^{\prime }\left(w^{T}Z\right)\right]w$, where g(·) is a nonlinear function, such as $g_{1}\left(y\right)=\tan h\left(a_{1}y\right)$, $g_{2}\left(y\right)=y\exp\left(-y^{2}/2\right)$ and $g_{3}\left(y\right)=y^{3}$;
(4) Normalize w by $w=w^{+}/\|w^{+}\|$;
(5) Go back to step “3” if not converged.

The vertical GPS coordinate time series are seen as the inputs of ICA and a set of ICs and the SRs can be obtained after ICA processing. Then we can obtain the model of common seasonal variations for the GPS time series ${\hat{D}}_{season}$ by

(1) $${\hat{D}}_{season}=\sum (\hat{IC}s\;\times \;SRs)$$

where ICs are the common independent components extracted from the GPS time series using ICA, and they are thought to represent the common seasonal sources after identification. SRs is the spatial response values in ICA separation. $\hat{IC}s$ are the modeled result of ICs and they are estimated using the VC regression method by the following equations:

(2) $$\hat{IC}s=a\left(t\right)\sin\left(2\pi t\right)+b\left(t\right)\cos\left(2\pi t\right)+c\left(t\right)\sin\left(4\pi t\right)+d\left(t\right)\cos\left(4\pi t\right)+\varepsilon$$

### 2.2. Varying Coefficient Regression Method

The expression of varying coefficient regression model is as follows [41]:

(3) $$Y=\beta_{1}\left(U\right)X_{1}+\beta_{2}\left(U\right)X_{2}+\cdots +\beta_{p}\left(U\right)X_{p}+\varepsilon$$

where Y is the variable to be fitted, $\;X_{i}$ is the regression variables, $\beta_{i}\left(U\right)$ is the coefficient which is a function of another variable U, and ε is the fitting error. When U is defined as a time variable, the Equation (1) would be an expression of temporal varying coefficient regression used in modeling the ICs in Equation (2). Here, we estimated the varying coefficients using local linear fitting [42]. For n groups of independent observed data, the sample form of the VC regression model is as follows:

(4) $$Y_{i}=\overset{p}{\underset{j=1}{\sum }}\beta_{j}\left(U_{i}\right)X_{ij}+\varepsilon_{i},\;\;i=1,2,\cdots ,\;n.$$

Assume $\beta_{j}\left(u\right)$ have continuous derivatives with respect to u, and the range of u is μ. According to Taylor’s formula, in the range of μ, for any given $u_{0}$, there exists:

(5) $$\beta_{j}\left(u\right)\approx \beta_{j}\left(u_{0}\right)+\beta_{j}^{\prime }\left(u_{0}\right)\left(u-u_{0}\right),\;\;j=1,2,\cdots ,\;p.$$

Define a kernel function as  Κ(t), and ${Κ}_{h}\left(t\right)=Κ\left(t/h\right)/h$. A Gauss kernel function was used in our following case study. The h is the window width parameter, and the determination of h will be introduced below. The local linear fitting of the coefficient in VC regression chooses $\beta_{j}\left(u_{0}\right)$ and $\beta_{j}^{\prime }\left(u_{0}\right)$ to satisfy the following condition:

(6) $$\overset{n}{\underset{i=1}{\sum }}{\left(Y_{i}-\overset{p}{\underset{i=1}{\sum }}\left(\beta_{j}\left(u_{0}\right)+\beta_{j}^{\prime }\left(u_{0}\right)\left(u-u_{0}\right)\right)X_{ij}\right)}^{2}{Κ}_{h}\left(t\right)\left(U_{i}-u_{0}\right)=min$$

Assuming:

$$X\left(u_{0}\right)=\left(\begin{array}{ccc} X_{11} & \cdots & X_{1p}\\ X_{21} & \cdots & X_{1p}\\ \begin{array}{c} \vdots \\ X_{n1} \end{array} & \begin{array}{c} \ddots \\ \cdots \end{array} & \begin{array}{c} \vdots \\ X_{np} \end{array} \end{array}\;\;\;\;\begin{array}{ccc} X_{11}\left(U_{1}-u_{0}\right) & \cdots & X_{1p}\left(U_{1}-u_{0}\right)\\ X_{21}\left(U_{2}-u_{0}\right) & \cdots & X_{2p}\left(U_{2}-u_{0}\right)\\ \begin{array}{c} \vdots \\ X_{n1}\left(U_{n}-u_{0}\right) \end{array} & \begin{array}{c} \ddots \\ \cdots \end{array} & \begin{array}{c} \vdots \\ X_{np}\left(U_{n}-u_{0}\right) \end{array} \end{array}\right),$$

$$Y={\left(Y_{1},Y_{2},\cdots ,\;Y_{n}\right)}^{T},$$

$$W\left(u_{0}\right)=\mathrm{Diag}\left({Κ}_{h}\left(U_{1}-u_{0}\right),{Κ}_{h}\left(U_{2}-u_{0}\right),\cdots ,{Κ}_{h}\left(U_{n}-u_{0}\right)\right),$$

$$a\left(u_{0}\right)={\left(\beta_{1}\left(u_{0}\right),\beta_{2}\left(u_{0}\right),\cdots ,\beta_{p}\left(u_{0}\right),\beta_{1}^{\prime }\left(u_{0}\right),\beta_{2}^{\prime }\left(u_{0}\right)\cdots ,\beta_{p}^{\prime }\left(u_{0}\right)\right)}^{T},$$

and the solution of the above weighted least squares situation can be expressed as:

(7) $$\begin{array}{c} \hat{a}\left(u_{0}\right)={\left({\hat{\beta }}_{1}\left(u_{0}\right),{\hat{\beta }}_{2}\left(u_{0}\right),\cdots ,{\hat{\beta }}_{p}\left(u_{0}\right),{\hat{\beta }}_{1}^{\prime }\left(u_{0}\right),{\hat{\beta }}_{2}^{\prime }\left(u_{0}\right)\cdots ,{\hat{\beta }}_{p}^{\prime }\left(u_{0}\right)\right)}^{T}\\ ={\left(X^{T}\left(u_{0}\right)W\left(u_{0}\right)X\left(u_{0}\right)\right)}^{-1}X^{T}\left(u_{0}\right)W\left(u_{0}\right)Y \end{array}$$

Thus, the local linear estimations of the coefficients β(u) at $u_{0}$ can be expressed as:

(8) $$\hat{\beta }\left(u_{0}\right)={\left({\hat{\beta }}_{1}\left(u_{0}\right),{\hat{\beta }}_{2}\left(u_{0}\right),\cdots ,{\hat{\beta }}_{p}\left(u_{0}\right)\right)}^{T}=\left(I_{p},0_{p}\right)\left({\left(X^{T}\left(u_{0}\right)W\left(u_{0}\right)X\left(u_{0}\right)\right)}^{-1}X^{T}\left(u_{0}\right)W\left(u_{0}\right)Y\right)$$

where $I_{p}$ and $0_{p}$ are the unit matrix and zero matrix with order p. We define $e_{j,2p}$ as a 2p-dimensional column vector with jth element being 1, and the remaining elements being 0, then we have:

(9) $${\hat{\beta }}_{j}\left(u_{0}\right)=e_{j,2p}^{T}\hat{a}\left(u_{0}\right),\;\;\;\;j=1,2,\cdots ,\;p.$$

The coefficient ${\hat{\beta }}_{j}\left(u_{0}\right)$ can be estimated as $u_{0}=U_{1},U_{2},\cdots ,U_{n}$ according to Equation (9), then we can calculate Y at each design point $U_{i}\left(i=1,\cdots ,n\right)$ according to Equation (8):

(10) $$\begin{array}{c} {\hat{Y}}_{i}=\left(X_{i1},\cdots ,X_{ip}\right){\left({\hat{\beta }}_{1}\left(U_{i}\right),\cdots ,{\hat{\beta }}_{p}\left(U_{i}\right)\right)}^{T}\\ =X_{i}^{T}\left(I_{p},0_{p}\right){\left(X^{T}\left(U_{i}\right)W\left(U_{i}\right)X\left(U_{i}\right)\right)}^{-1}X^{T}\left(U_{i}\right)W\left(U_{i}\right)Y\\ =\left(X_{i}^{T},0_{1\times p}\right){\left(X^{T}\left(U_{i}\right)W\left(U_{i}\right)X\left(U_{i}\right)\right)}^{-1}X^{T}\left(U_{i}\right)W\left(U_{i}\right)Y \end{array}$$

where $X_{i}^{T}=\left(X_{i1},\cdots ,X_{ip}\right)$ is the ith variables of $X_{1},\cdots ,X_{p}$. The final result of Equation (10) can be expressed in the style of $\hat{Y}=LY$, where L **is** called the pseudo-hat matrix.

We used the cross-confirmation (CV) method to select the best window width parameter. Taking the range of the window width to be selected for a given step length, with every h within the range, we can obtain the fitting results $\hat{Y}=L\left(h\right)Y$ using a local linear fitting method, where L(h) represents the pseudo-hat matrix with series selected h. The process of cross-validation is as follows: for a given window width parameter  h, except for the ith variable $\;\left(Y_{i},X_{i}\right)$, we can calculate the fitting value of the regression function at $X_{i}$ for the other n−1 data using the local linear fitting method under the given h, defined as:

(11) $$CV\left(h\right)=\frac{1}{n}\overset{n}{\underset{i=1}{\sum }}{\left(Y_{i}-{\hat{Y}}_{\left(-i\right)}\left(h\right)\right)}^{2}$$

The CV(h) values of each h were calculated and h corresponds to the minimum value of CV(h) which is the optimal window width parameter. 

## 3. Case Study

### 3.1. GPS Data 

**Figure 2:**
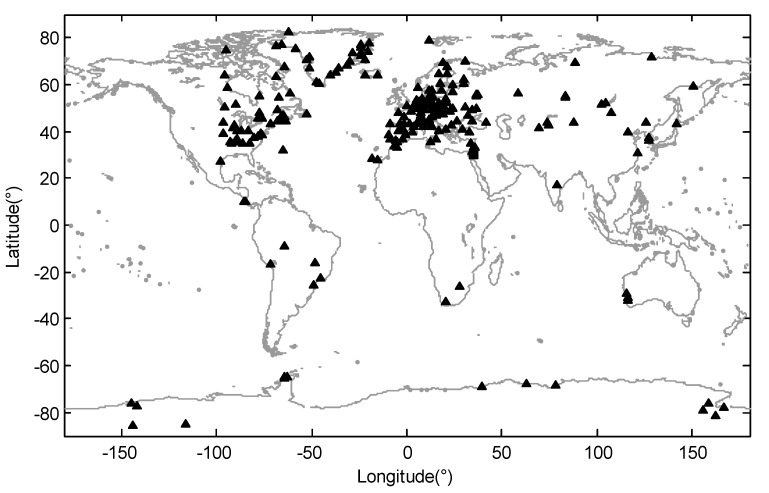
Distribution of Global Positioning System (GPS) sites used in the case study.

The International GPS Service for Geodynamics (IGS) GPS time series processed by the Scripps Orbit and Permanent Array Center (SOPAC) and California Spatial Reference Center (CSRC) were used in our case study (available at http://garner.ucsd.edu/). The outliers and trend item have been removed in the time series products. To perform the ICA analysis, we completed further processing on the vertical GPS time series. We chose GPS time series data which span the eight years from the beginning of 2010 to the end of 2017. Spatiotemporal analysis with ICA works most effectively with data that have few gaps. As such, we only used sites with more than 70% data. Additionally, we removed the sites with obvious offset in the time series using manual methods [43] and the obvious abnormal sites, which would affect the separation of common seasonal ICs. To identify the “abnormal” sites, we computed the correlation coefficients between the series at each site and the series at the four nearest sites. If the correlation coefficients were all less than 0.4, we considered the time series at this site as being affected by local geophysical deformation or equipment changes, and it would be defined as an abnormal site and removed. Finally, a total of 262 GPS sites around the world were chosen in the case study, and the distribution of the sites are shown in Figure 2. After this, a kriged kalman filter technique was used to interpolate the missing data of the 262 vertical GPS time series [44]. We did not complete additional processing in the correction of other potential seasonal signals, including the draconic year effect and bedrock thermal expansion, although we think the mass loadings are the main contribution to the seasonal variation in vertical GPS coordinate time series. Because in the separation of ICA, as long as the existing source signals are statistically independent, the separation of independent components will not be obviously affected by the existence of errors.

### 3.2. Spatiotemporal Analysis Using ICA

**Figure 3:**
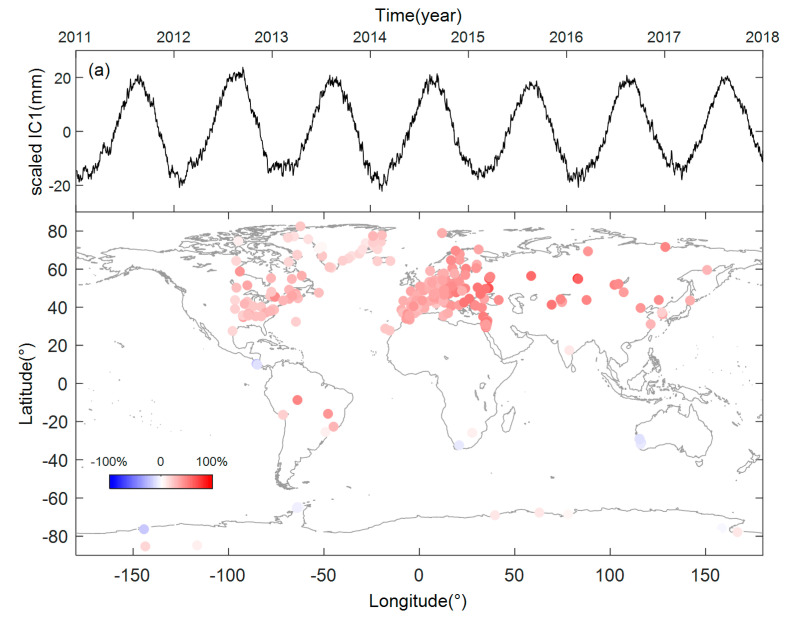

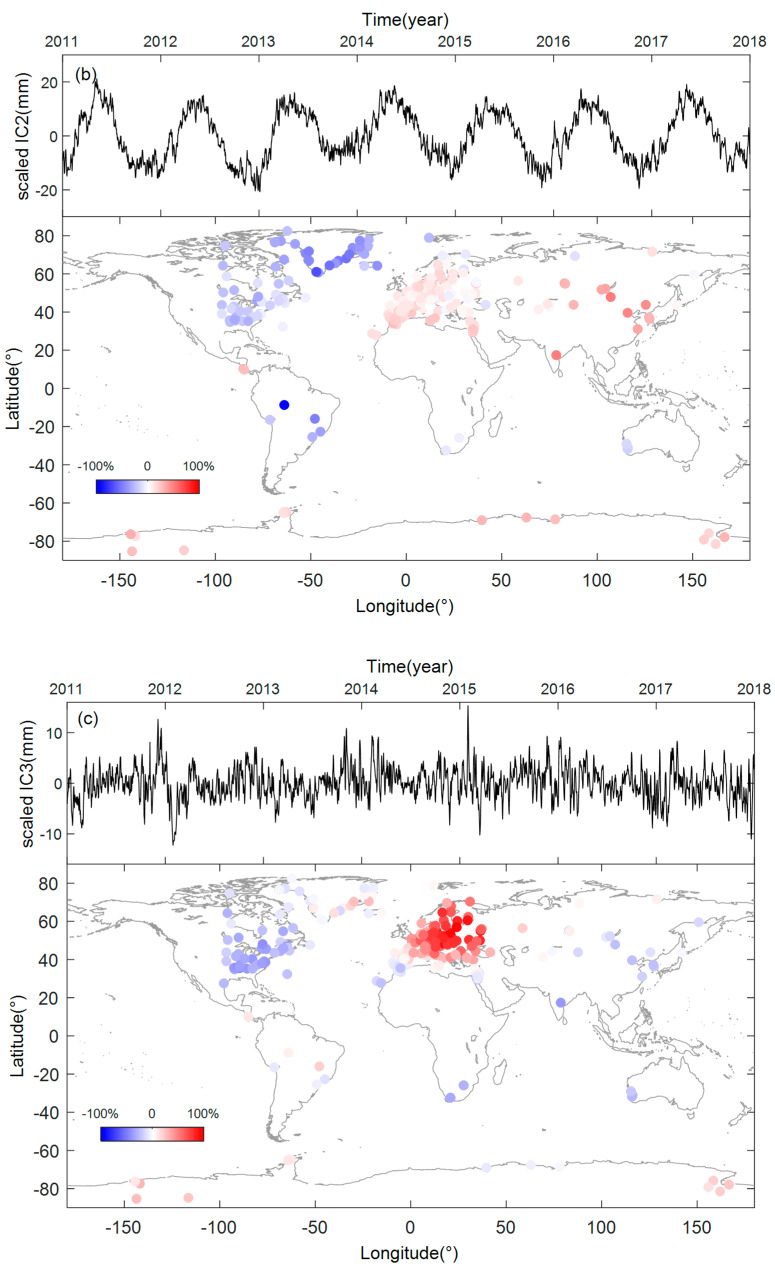

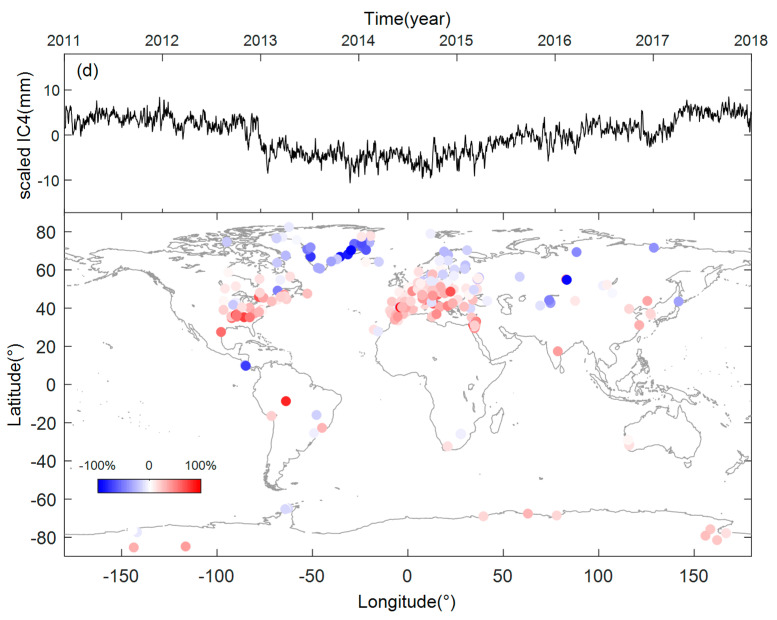
Top 4 Scaled independent components (ICs) and the spatial responses (SRs). (**a**) scaled IC1 and SR1. (**b**) scaled IC2 and SR2. (**c**) scaled IC3 and SR3. (**d**) scaled IC4 and SR4.

We acquired 262 sets of complete vertical GPS time series after the selection and processing steps. PCA was used to preprocess the GPS time series before we performed ICA. An appropriate number of PCs need to remain for the following ICA processing. We completed several tests and found that in most cases the choice of the number of remaining PCs had little effect on the separation of the top four ICs. In our study, the top six principle components with more than 80% contribution of variance were retained and used in the following analysis. Then, FastICA was used to separate the ICs from the mixtures, and we received a series of temporal components and their corresponding spatial patterns. Unlike PCA, the order of ICs is random in ICA and we rearranged the ICs according to their contributions based on their ratio values, defined by Liu et al. [33]. To enable comparisons, we divided each spatial response of the independent component by the maximum of its absolute value and the corresponding ICs are multiplied by the scale. As a result, all the spatial patterns are in the range of -100% to 100%. The top four scaled ICs and their spatial responses are shown in Figure 3. 

Note the temporal components in Figure 3a,b, the top two ICs have obvious seasonal signals. Especially for the first independent component, we can see a significant annual period signal and this shows consistent positive spatial responses to almost all of the global GPS sites. We noticed that the SRs of IC1 are most obvious for the sites in northern Asia, northeastern Mediterranean and the area near the Black Sea, but relatively small for the sites in midwestern Europe and North America. The SRs of IC1 are the least obvious for most of the sites in Greenland, Antarctica and the two sites on the west coast of Australia, while the two sites near the Amazon basin in South America have more obvious spatial responses of IC1. IC1 may be a response of common hydrological mass variation.

IC2 and its spatial responses are shown in Figure 3b. It shows seasonal variations, and obvious differences exist in the annual amplitudes, which cannot be described using constant sine and cosine functions. The SRs of IC2 are most obvious for the sites in Greenland, followed by the sites in the Americas, and are least obvious for the sites in west Asia and Europe. The spatial responses of IC2 show special distribution, that is, apart from the sites on the Antarctic Peninsula, IC2 shows obvious opposite responses to the sites in the eastern and western hemispheres. IC2 may be driven by climate or some other environmental factors.

The spatial and temporal patterns of IC3 and IC4 are shown in Figure 3c,d. IC3 looks like a random error series and has opposite responses to the sites in Europe and other regions, especially for the sites in eastern Europe and North America, where this contrast is most obvious. The source of IC3 needs to be further explored. IC4 shows long-term trend changes, which describe the nonlinear long-tern trend change in the vertical GPS time series. We notice that this trend is most obvious in the Greenland site, and it may be related to the short-term ice mass change in Greenland during the period. Considering the negative spatial responses, most GPS sites in Greenland show uplift from early 2011 to 2013 and this is followed by a subsidence from 2014 to 2017, which is consistent with the transient ice mass changes during the period [45,46]. 

### 3.3. Modeling Common Seasonal Signals 

**Figure 4:**
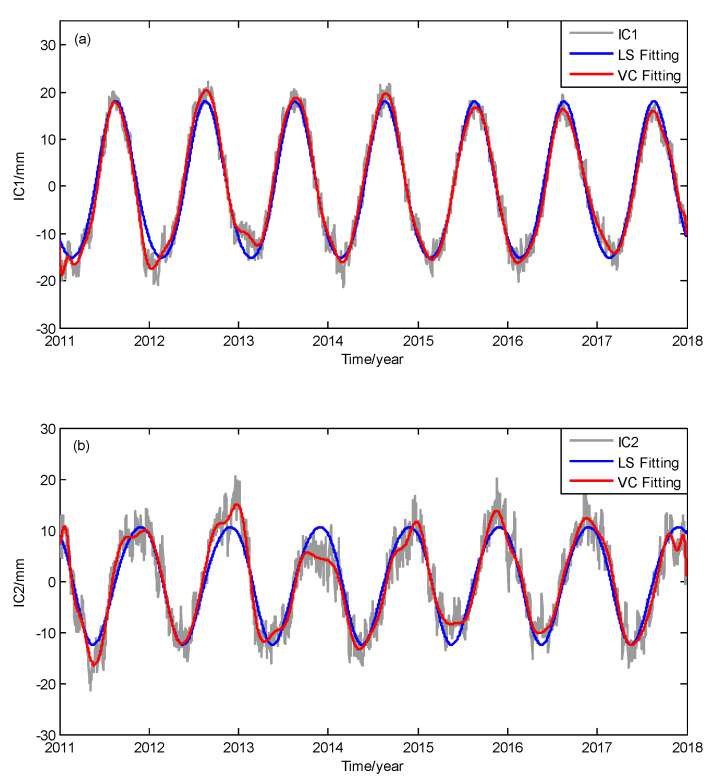
Fitting results of (**a**) IC1 and (**b**) IC2 using least squares (LS) regression and varying coefficient (VC) regression.

After the spatiotemporal filtering of vertical GPS time series, the top two ICs are thought to represent the common seasonal signals for all the sites. Thus, the next step is modeling the IC1 and IC2 using a varying coefficient regression method based on Equation (2). As comparisons, the least square regression method (LS) is also used to fit IC1 and IC2. The fitting results for ICs are shown in Figure 4.

**Figure 5:**
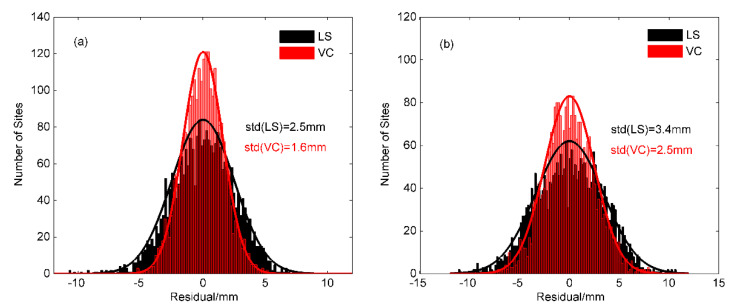
Histogram of Residual in the fitting model of least squares regression (LS) and varying coefficient regression (VC) for (**a**) IC1 and (**b**) IC2, the std of residual are marked.

As shown in Figure 4, both LS and VC regression models fit the periodic variations of IC1 and IC2. However, the annual amplitudes are time varying for the ICs, especially for IC2 in Figure 4b. We notice that the LS regression can only achieve constant periodic fitting results, both in Figure 4a,b, while the VC regression method can closely fit the seasonal changes in each year, with better flexibility and adaptability. The histogram of residuals of different methods are shown in Figure 5. All the residuals present normal distributions and note that the residuals of VC regression for IC1 and IC2 are more concentrated. The standard deviation (std) values of IC1 fitting residuals using the LS and VC regression models are 2.5 mm and 1.6 mm, respectively, and the std values of IC2 fitting residuals using the LS and VC regression models are 3.4 mm and 2.5 mm, respectively. 

### 3.4. Spatiotemporal Modeling and Discussion 

**Figure 6:**
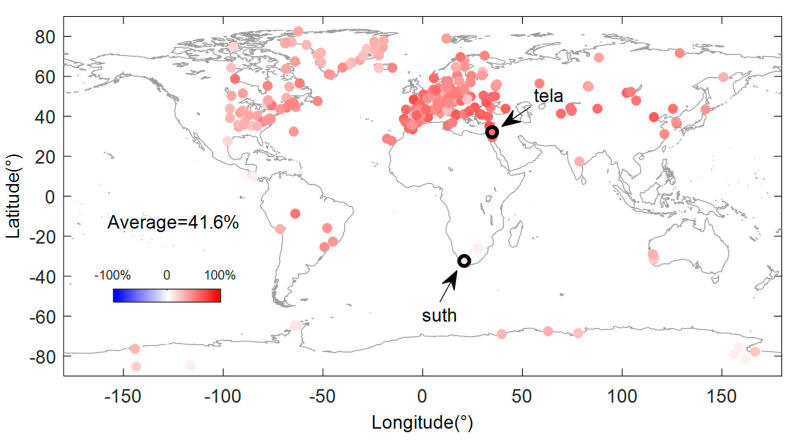
The root mean square (RMS) reduction in coordinate time series after the model correction for each site, the sites “tela” and “suth” are marked.

We obtained the model of IC1 and IC2 using the varying coefficient regression, which is thought to represent the main common seasonal changes in the vertical GPS time series in our case study. According to Equation (1), by multiplying by the spatial responses we obtained the spatiotemporal model of the main common seasonal signals ${\hat{D}}_{season}$:

(12) $${\hat{D}}_{season}=\hat{IC}1\;\times \;SR2+\hat{IC}2\;\times \;SR2$$

where $\hat{IC}1$ and $\hat{IC}2$ are the modeling results of IC1 and IC2 using VC regression. The window width parameter h in the VC regression is selected as 39.4 through a cross-confirmation method. We then removed the seasonal model from al the vertical GPS time series and obtained the seasonal corrected series. The correction effects were evaluated using Equation (13). The $RMS_{reduction}$ values for all the sites are shown in Figure 6.

(13) $$RMS_{reduction}=\frac{RMS_{GPS}-RMS_{GPS-\hat{D}\left(season\right)}}{RMS_{GPS}}$$

**Figure 7:**
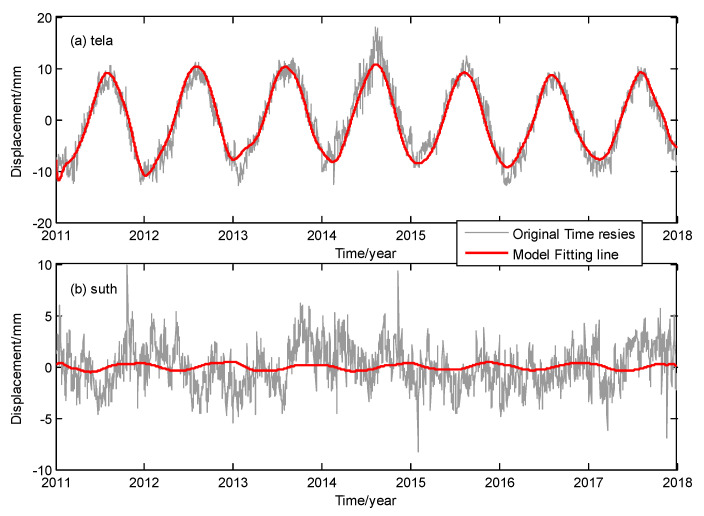
Vertical coordinate time series and the fitting lines of spatiotemporal model for sites (**a**) “tela” and (**b**) “suth”.

It can be seen that the $\;RMS_{reduction}$ values of all the GPS time series have been reduced by varying degrees after the model correction, with an average correction effect of 41.6%, and the average correction effect decreases to 41.2% when using LS regression to model the ICs. Among them, the most obvious seasonal corrections occurred at the sites in the Eurasian region, followed by the sites in the Americas and Greenland. The corrections effects of several sites in Africa, Australia and Antarctica were the worst. This is related to the insignificant seasonality of these sites. The site with the best correction effect is "tela”, with an improvement of 71.8%, and the lowest improvement rate is only 0.7% at the site ”suth”. The two sites were marked in Figure 6. The fitting effect of the GPS time series of sites “tela” and “suth” are shown in Figure 7. Note that the site “suth” is located in southern Africa, and seasonal variation in its time series is insignificant (Figure 7). The top two ICs did not separate the non-obvious nonlinear changes. Therefore, the fitting effect of the spatiotemporal model is limited for this site. 

**Figure 8:**
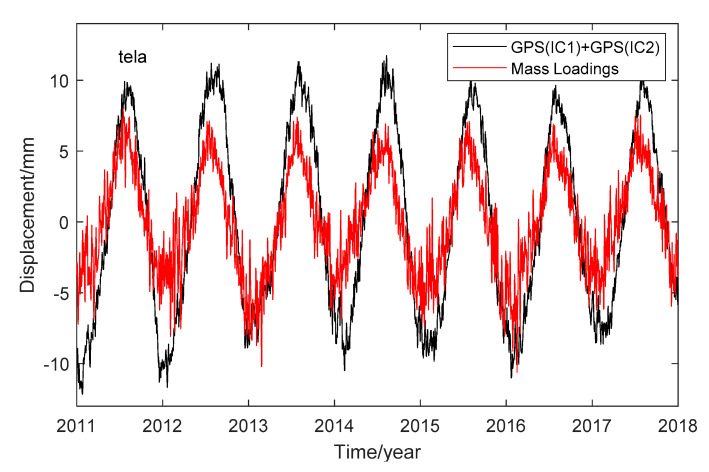
Comparison between ICs derived displacements (GPS(ICs)) and mass loading displacements at site “tela”.

To explore the geophysical sources of the IC1 and IC2, we took the site “tela” as an example. We computed mass loading displacements at this site via the International Mass Loading Service [18], including the atmospheric loading (ATML), land water storage mass loading (LWS) and non-tidal loading (NTOL). For ATML and LWS, we use the model of Modern Era Retrospective-analysis for Research and Applications (MERRA2), and for NOTL we use the model of the Ocean Model for Circulation and Tides (OMCT). We averaged the loading series into daily results to make comparisons with the ICs derived displacements (GPS(ICs)). We found the mass loadings are not consistent with the individual ICs, but the sum of mass loadings is highly correlated with the sum of GPS(IC1) and GPS(IC2), with a correlation coefficient of 0.87. The comparison between their displacements is shown in Figure 8. We find that the amplitude of the mass loadings is less than that of GPS(ICs), which indicates the mass loading may be underestimated at the site ”tela”. We further corrected the mass loading effects from the GPS time series at the site “tela” and the $RMS_{reduction}$ is 40.4%, while for the conditions of VC and LS regression fitting for ICs, the correction effect is 71.8% and 68.4%, respectively.

## 4. Conclusions

We propose a spatiotemporal model based on ICA and VC regression methods to fit the common seasonal signals in vertical GPS coordinate time series. On the basis of ICA spatiotemporal analysis, a varying coefficient regression method was used to model the common independent seasonal signals, then, combined with the spatial responses of ICs, the fitting model of the seasonal variations for all the vertical time series can be achieved. The use of ICA makes it possible that by modeling a few temporal components, we can achieve the fitting of the seasonal changes in the time series of all GPS sites. The use of VC regression solves the problem that the least squares regression cannot accurately fit the time varying period signals. 

The spatiotemporal modeling method was then used in the fitting of seasonal signals in the vertical time series of 262 GPS sites worldwide. We separated several independent components from the GPS time series and analyzed the possible geophysical origin based on their temporal and spatial characteristics. We used VC regression to fit the top two ICs and the results show that VC regression performs a better fitting to the LS regression. We obtained the spatiotemporal model of the common seasonal signals in all the 262 GPS sites and corrected the modeling results from original GPS time series. The RMS values for all the GPS time series have been reduced after seasonal signals correction, with an average reduction of 41.6%. It is also noticed that our proposed spatiotemporal model mainly accounts for the common seasonal variations at the sites, and the local seasonal signals that exist in individual sites are not reflected in the common mode ICs. For the GPS time series contained with local seasonal responses, a better choice would be to model the coordinate time series separately using the varying coefficient regression method.
